# Tumor organoids for cancer research and personalized medicine

**DOI:** 10.20892/j.issn.2095-3941.2021.0335

**Published:** 2021-09-14

**Authors:** Hui Yang, Yinuo Wang, Peng Wang, Ning Zhang, Pengyuan Wang

**Affiliations:** 1 Translational Cancer Research Center, Peking University First Hospital, Beijing 100034, China; 2 Division of General Surgery, Peking University First Hospital, Beijing 100034, China

**Keywords:** Organoids, cancer research, heterogeneity, personalized medicine, clinical cancer therapy

## Abstract

Organoids are three-dimensional culture systems generated from embryonic stem cells, induced pluripotent stem cells, and adult stem cells. They are capable of cell proliferation, differentiation, and self-renewal. Upon stimulation by signal factors and/or growth factors, organoids self-assemble to replicate the morphological and structural characteristics of the corresponding organs. They provide an extraordinary platform for investigating organ development and mimicking pathological processes. Organoid biobanks derived from a wide range of carcinomas have been established to represent different lesions or stages of clinical tumors. Importantly, genomic and transcriptomic analyses have confirmed maintenance of intra- and interpatient heterogeneities in organoids. Therefore, this technology has the potential to revolutionize drug screening and personalized medicine. In this review, we summarized the characteristics and applications of organoids in cancer research by the establishment of organoid biobanks directly from tumor organoids or from genetically modified non-cancerous organoids. We also analyzed the current state of organoid applications in drug screening and personalized medicine.

## Introduction

Cancer is the leading cause of death among noncommunicable diseases^1^ and the survivals of several cancers remain extremely low^2^. One of the reasons is a shortage of suitable preclinical tumor models, which hinders the development of anti-cancer drugs and precision treatments^3^. Existing preclinical models rely mainly on cancer cell lines and patient-derived xenografts (PDXs). Cancer cell lines, the earliest and most widely used tool in tumor research, are characterized by high manipulability, short culture cycle, and high throughput^4^. However, they lack the phenotype and genetic heterogeneity of the original tumor^5^. In comparison, PDX models preserve the structures, cell compositions, molecular characteristics, and complexities of the original tumors^6,7^, but are costly and time-consuming. Moreover, given that they are usually derived from a small number of tumor cells, they may not capture the original tumor’s heterogeneity^8^. Therefore, in recent years, cancer research has focused on new three-dimensional (3D) culture models. In 2009, Sato et al.^9^ used Lgr5+ stem cells from the bottom of small intestinal crypts to generate villus-like epithelial domains by culturing the cells in Matrigel with niche factors R-spondin, epidermal growth factor (EGF), noggin, and Wnt. This strategy yielded an organ-like structure, termed “organoid”^9^ and defined the starting point for organoid culturing of multiple mouse and human epithelia, as well as tumor-derived organoids.

Organoids are defined as a collection of organ-specific cell types produced from organ progenitors or stem cells that self-organize into the corresponding organ *via* 3D culture^10^. Compared to other preclinical models, they are superior in copying the phenotypes of the organs and reproducing the genetic heterogeneities of the original tumors^11^. In the 5 years following their discoveries, organoid models of the colon and small intestine^12,13^, retina^14^, brain^14^, liver^15^, stomach^16–18^ and mammary glands^19^ were successfully cultured. Gao et al.^20^ introduced organoid models for cancer research in 2014 by establishing systems with metastatic tissues and circulating tumor cells harvested from patients with advanced prostate cancers. Next, organoid models of colon carcinomas^21^, prostate carcinomas^22^, pancreatic carcinomas^23,24^, renal carcinomas^25^, gastric carcinomas^26^, breast carcinomas^27^, ovarian carcinomas^28^, primary liver carcinomas^29^, and bladder carcinomas^30^ were established. “Growing organoids” was selected as the “Breakthrough of the Year” by *Science* in 2013^31^ and “Method of the Year” by *Nature Methods* in 2017^32^. In 2018, Vlachogiannis et al.^33^ demonstrated the potential of organoids to predict patient clinical outcomes, suggesting their implementation in personalized medicine. Taken together, organoids provide a reliable method for preclinical disease modeling and drug screening, providing the bases for gene and stem cell therapies for cancer.

## The development of organoid culture methods

Over the past decades, stem cells and/or progenitor cells have been used to reconstruct organs because of their capacities for self-renewal and differentiation into tissue-specific lineages^10,34^. Moreover, regenerative medicine has highlighted the ability of stem cells to differentiate into ≥ 1 cell types to repair damaged organs^10,35,36^. Eiraku et al.^37,38^ demonstrated the ability of cortical tissue to form complex optical cup structures *in vitro*, owing to the self-organizing property of pluripotent stem cells. Sato et al.^9^ showed that adult intestinal stem cells formed the main structures of intestinal organs, and the organoids could be propagated in culture for extended periods of time.

In recent years, organoids have been generated from tissue samples containing embryonic stem cells, induced pluripotent stem cells, and adult stem cells^39,40^. Embryonic stem cell-derived organoids have the advantage of simulating the morphological traits of organ development and transplantation *in vivo*; whereas organoids derived from the other 2 stem cell types play a fundamental role in precision medicine for modeling and drug screening of refractory diseases^39^.

Organoids are currently cultivated using a solid extracellular matrix to support cell proliferation and adhesion^41^; as well as animal-derived hydrogels, including Matrigel^42^ and collagen^43^, to promote organoid structure^44^. For example, Lgr5+ stem cells or crypts were coated in Matrigel and then cultured in medium containing EGF, R-spondin, and noggin to generate intestinal organoids^9,45^.

Recently, it has been reported that Matrigel-based cultivation might limit organoid growth and maturation by restricting gas and metabolite exchanges between organoids and the surrounding microenvironment^46^. Therefore, Matrigel has been replaced with sponge-like^47^ or fibrous reticular scaffolds^48^, which contain larger cavities. Robertson et al.^49^ used the internal vascular network of the liver to facilitate better transport to and from liver organoids, and thus increased their *in vitro* survival beyond 4 weeks. This breakthrough represented an important development for organoid generation and culture.

**Figure 1:**
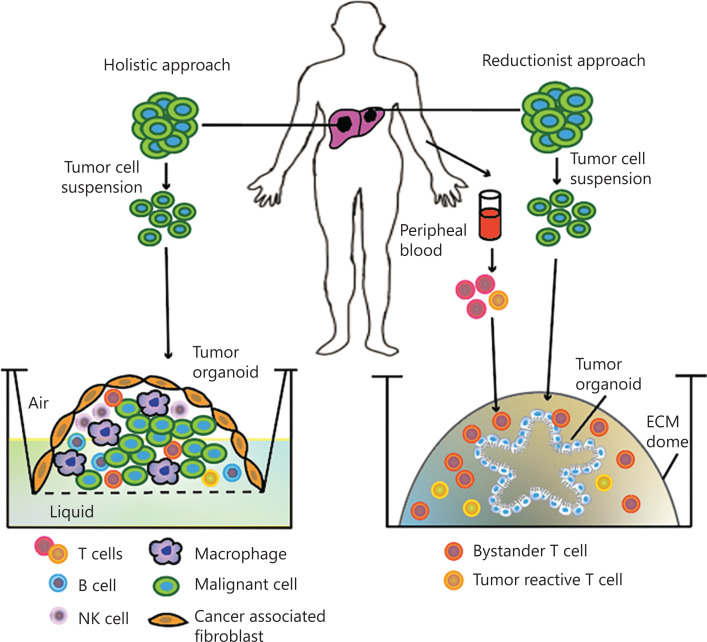
Tumor-immune co-cultured organoid systems. In the holistic approach (left), a tumor cell suspension is cultured in an air-liquid interphase including all tumor cell types, endogenous immune cells, and fibroblasts, to generate tumor-immune organoids, facilitating culture of the entire tumor microenvironment, which closely resembles the *in vivo* situation. In the reductionist approach (right), tumor organoids are grown and expanded from a tumor cell suspension and then co-cultured with autologous immune cells from the peripheral blood of the same patient to promote the serial expansion of tumor-reactive T cells, which enables more extended investigations. ECM, extracellular matrix.

There is a growing interest in constructing a tumor-immune co-cultured organoid system that can preserve the tumor microenvironment^50,51^ (**Figure 1**). Several studies using a holistic^52–54^ or reductionist approach^55^ to build tumor-immune co-cultured *ex vivo* 3D models have been published. Neal et al.^52^ reported an air-liquid interface murine organoid model containing tightly integrated epithelial and stromal compartments, as well as specific tumor-infiltrating lymphocytes. Besides basal media, additional T cell activators such as inteleukin-2 were added to support the growth of immune cells. Consequently, variable subtypes, such as CD8+ T cells, CD4+ T cells, B cells, natural killer cells, and natural killer T cells, could be preserved over a period of days. In the reductionist approach, organoids are grown from tumor biopsies and are then co-cultured with autologous immune cells from the peripheral blood of the same patient to promote the serial expansion of tumor-reactive cells, allowing for the long-term culture and growth of the tumor epithelium (**Figure 1**)^52^. These co-cultured organoid systems enable investigations within the tumor microenvironment and facilitate personalized immunotherapy testing.

## Organoid techniques facilitate research in developmental and cancer biology

Organoids are 3D cell cultures with unique biological characteristics, such as proliferation and differentiation into multifunctional cells under specific developmental conditions^56^ as well as self-assembly to mimic the structure of the parental organ^57,58^. In the case of primary liver cancer, Broutier et al.^29^ established organoids from hepatocellular carcinomas (HCCs), combined hepatocellular-cholangiocarcinomas (CHCs), and cholangiocarcinomas (CCs). Histological analysis demonstrated that HCC-derived organoids exhibited pseudoglandular rosettes as their parental tissues, whereas CC-derived organoids presented glandular lumens similar to the clinical morphology of the original patient, indicating genetic stability even after long-term expansion^29^. Moreover, organoids can recapitulate the physiological activity of the corresponding organs^40,59^. Huch et al.^15^ showed that, similar to primary hepatocytes, organoids from hepatocytes secreted albumin. In addition, organoids can mimic filtration, neural activity, and contraction processes^10^. Genomic and transcriptomic profiles have been investigated for a wide range of cancer-derived organoids. Although limited tumor evolution has been reported in organoid cultures, for the most part, profiles were similar between samples within individual organoid lines, indicating that tumor heterogeneity was well preserved^30^.

Based on these characteristics, basic research has been revolutionized by organoid technology, providing new possibilities for the study of development and organogenesis^41^. Organogenesis is a complex and interconnected process orchestrated *via* interactions across boundaries^60,61^. Its study has been limited by the availability of embryonic or fetal tissues and ethical concerns^41^. Organoids have helped demonstrate how individual neighboring tissues coordinate the onset of multi-organ structures^62^. Koike et al.^62^ reported that the anterior and posterior gut spheroids, differentiated from 3D-cultured human pluripotent stem cells, could interact with each other and form hepatic, biliary, and pancreatic organoids, recapitulating early morphogenetic events, such as invagination and branching of interconnected organ structures. Hence, organoids might offer an accessible model for the exploration of organogenesis and development.

**Figure 2:**
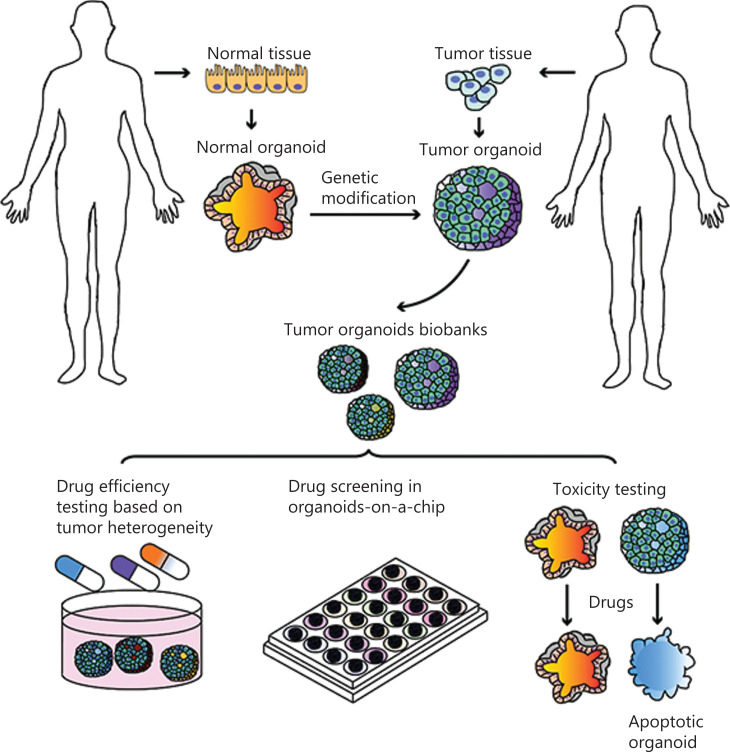
Organoids in cancer research and personalized medicine. Tumor organoid biobanks can be established from genetically modified noncancerous organoids or tumor organoids. These biobanks enable drug efficacy testing, drug screening, and toxicity testing, to facilitate personalized clinical medicine.

Organoids are of great importance in cancer research and personalized medicine. Theoretically, organoids allow the expansion of samples derived from tumor tissues of individual patients with a range of carcinomas^63^. Researchers have demonstrated that organoids can be established from surgical specimens^63^, fine-needle aspiration, and even from ascites liquid^64^, providing alternative sources of different organoid lines. In addition, CRISPR-based genetic modifications allow the engineering of non-cancerous organoids by introducing oncogenic alterations, providing ways to investigate tumor initiation and progression, which is vital in cancer research and personalized medicine. In this review, we analyzed the various applications of tumor organoids by using tumor biobanks and known genetic modifications of organoids, and summarized existing uses of organoids for personalized medicine (**Figure 2**).

## Development of tumor organoid biobanks

Tumor organoid biobanks are generated in 2 ways: *via* a direct tissue collection from patients by biopsy or surgical resection^65–67^ or modification of organoids derived from healthy tissues^68,69^. Tumor organoid biobanks allow passage, expansion, and cryopreservation of organoids derived from different carcinomas, as well as different lesions, grades, or stages of an indicated cancer. Established tumor organoid biobanks are listed in **Table 1**.

**Table 1:** Overview of tumor organoid tissue banks

Tumor organoid tissue banks	Source	No. of patients involved	No. of tumor organoid cell lines	Efficiency	Reference
Prostate cancer	Metastases and circulating tumor cells	7	7	∼15%–20%	^20^
Colorectal cancer	Primary tumor	20	22	∼90%	^70^
	Metastases	14	10	71%	^67^
	Primary tumor and metastases	43	55	100%	^71^
Breast cancer	Primary tumor	>100	>150	∼80%	^27^
Gastric cancer	Primary tumor and metastases	34	46	>50%	^72^
	Metastatic gastrointestinal cancer	71	77	70%	^33^
Bladder cancer	Primary tumor	16	12	∼70%	^30^
Pancreatic cancer	Primary tumor and ascites specimens	49	39	∼80%	^64^
Ovarian cancer	Primary tumor	32	56	65%	^28^
Glioblastoma	Primary tumor	53	70	91.4%	^73^
Liver cancer	Primary tumor	8	7	Not reported	^29^
	Primary tumor	8	10	26%	^65^
	Primary tumor	5	27	Not reported	^74^

## Tumor organoid biobanks can be established from tissue collection

### The prostate cancer organoid biobank

The prostate cancer organoid biobank was established in 2014, introducing the use of organoids in cancer research^20^. Gao et al.^20^ cultured 6 organoid lines from prostate cancer metastatic lesions *via* tissue biopsy and 1 line from circulating tumor cells, resulting in 15%–20% efficiency. This small biobank included various subtypes of prostate cancers, such as high grade adenocarcinomas, mucinous adenocarcinomas, adenocarcinomas with extensive squamous differentiation, and adenocarcinomas with cribriform growth, which were consistent with the original patients. The histological and immunohistological patterns displayed in prostate cancer organoids were similar to those of the original patients. For example, organoids derived from bone marrow metastasis lesions recapitulated the intraductal pattern of primary cancer, with positive pan-cytokeratin and negative androgen receptor immunohistochemical staining. Furthermore, mRNA and protein analyses revealed that these organoid lines recapitulated the diversity observed in human prostate cancer^20^.

### The colorectal cancer organoid biobank

Since their first establishment, organoids originating from the small intestine have been expanded to also generate colorectal carcinoma organoids^9^. Barker et al.^75^ reported the *in vitro* formation of germinal cystic organ-like structures derived from mice with intestinal adenomas. Fumagalli et al.^76^ analyzed the evolution of adenomas *in vivo* using organoids containing different combinations of colorectal cancer mutations. The results indicated gene changes in Wnt, EGF receptor, TP53, and transforming growth factor-beta signaling pathways, which led to tumor growth, migration, and metastasis.

Wetering et al.^70^ reported the establishment of 22 cancer organoids along with 19 organoids derived from adjacent non-cancerous tissues from 20 colorectal carcinoma patients^70^. The organoids were successfully established in ∼90% of cases, while >80% of them could be successfully frozen and thawed. Patient-specific morphologies such as cystic *vs.* solid organization of the epithelium were generally preserved, and expressions of colorectal cancer markers (*Ki-67*, *OLFM4*, and *KRT20*) in organoids reflected those in the patients.

To generate different subtypes of colorectal cancer organoids, Fujii et al.^71^ improved organoid culture methods based on niche factor requirements. They established 55 colorectal cancer organoids derived from a range of histological subtypes and clinical stages, as well as 41 counterpart organoids from noncancerous colorectal tissues, including hyperplastic polyps, sessile serrated adenoma/polyps, early cancers, advanced cancers, and metastatic cancer subtypes. By carefully adjusting ambivalent factors, Wnt activators, and other inhibitors, they were able to propagate colorectal carcinoma organoids with 100% efficiency. All these organoids preserved the histopathological structures and profiles of the parental tumors^71^.

The methods involved in establishing colorectal carcinoma biobanks facilitated the generation of different tumor subtypes and promoted the creation of individual models for personalized medicine. Since then, organoids derived from a wide range of tumor subtypes have been established in multiple cancers.

### The breast cancer organoid biobank

Breast cancer comprises multiple pathologically and clinically distinct subtypes. Sachs et al.^27^ established 95 breast cancer organoids from 155 breast cancer specimens, with a success rate >80%. This organoid biobank included different histological subtypes, grades, and receptor statuses of the original tumor. Breast cancer-derived organoids showed a similar distribution of subtypes as clinical patients, i.e., 50%–80% invasive ductal carcinomas and 5%–15% invasive lobular carcinomas. These organoids presented significantly different morphologies, including sizes and cystic-like or grape-like phenotypes, in accordance with the original patients. Moreover, whole-genome sequencing (WGS) and RNA sequencing demonstrated that copy number variations, sequence changes, and expression profiling were similar to parental tumors even after long-term culture^27^.

### The gastric cancer organoid biobank

Yan et al.^72^ reported the establishment of a primary gastric cancer organoid biobank, including noncancerous tissues, dysplastic tissues, primary tumors, and lymph node metastases from 63 specimens of 34 patients. The establishment percentage was >90% in noncancerous gastric organoids and >50% in cancer organoids^72^. The biobank encompassed most known molecular subtypes, including microsatellite instability, Epstein-Barr virus, intestinal (chromosome instability), and diffuse (genomically stable) subtypes. The subtypes displayed a variety of growth patterns, such as solid, glandular, or discohesive patterns, illustrating different degrees of gastric carcinomas. Protein expression analyses confirmed the overexpressions of known oncogene markers, such as HER2, ERBB2, and FGFR2, in these cancer organoids^72^.

In the same year, Vlachogiannis et al.^33^ generated an organoid biobank using 110 metastatic gastrointestinal cancer specimens from 71 patients enrolled in phase I or II clinical trials. In this case, organoids were successfully established in 70% of the specimens. After confirming the phenotypic and genotypic characteristics of organoids with patient tumors, they focused on the application of organoids in drug screening. This study highlighted how patient-derived organoids could be used to recapitulate patient responses in the clinic, which might have potential applications in personalized medicine^33^.

### The bladder cancer organoid biobank

Lee et al.^30^ described an organoid biobank that recapitulated the histopathological and molecular diversities of human bladder cancers^30^. They cultured 22 bladder cancer organoids from 16 patients with an efficiency of ∼70%. The organoid morphology ranged from spheroidal to asymmetric, and 1 organoid line displayed features of squamous cell carcinoma, an uncommon histological subtype of bladder cancer similar to the parental tissue. These findings suggested the presence of multiple phenotypes in the bladder cancer organoid biobank. The biobank consisted of organoids collected directly from patients, serially passed organoids, organoids plated in orthotopic xenografts, and xenograft-derived organoids^30^. Using whole-genome and targeted exome sequencing, the organoids were found to retain parental tumor heterogeneity and exhibited a spectrum of genomic changes consistent with tumor evolution^30^.

### The pancreatic cancer organoid biobank

Pancreatic cancer is characterized by elevated intra- and inter-tumor heterogeneities and highly malignant phenotypes, which makes it difficult to establish organoid biobanks. In this respect, a significant contribution was made by Boj et al.^24^ and Seino et al.^64^.

Boj et al.^24^ first established organoid models using non-cancerous human pancreatic tissues, as well as neoplastic murine and human pancreatic tissues. These organoids exhibited ductal- and disease-stage-specific characteristics^24^. Seino et al.^64^ established a human pancreatic cancer organoid biobank using surgical, fine needle aspiration, and ascites specimens. Their pancreatic cancer organoid library was comprised of 39 pancreatic ductal adenocarcinoma (PDAC) patient-derived organoids. They identified 3 functional subtypes according to stem cell niche factor dependency on Wnt or R-spondin, revealing functional heterogeneity in tumor progression. They also collected surrounding cancer-associated fibroblasts, which provided a Wnt niche for PDAC, and thus obtained a platform for investigating the tumor microenvironment using organoids^64^.

### The ovarian cancer organoid biobank

Ovarian cancer is a heterogeneous disease with multiple subtypes. Kopper et al.^28^ established 56 organoid lines from 32 patients with a success of 65%, which represented all main subtypes of ovarian cancers, including serous borderline tumors (BT), mucinous BT, low grade serous BT, mucinous carcinomas, endometrioid, clear cell carcinomas, and the high grade serous subtypes. Consistent with the original patients, ovarian cancer organoids in this library displayed wide morphological variations between distinct histological subtypes. For example, most BT organoids were cystic; whereas mucinous carcinoma, low grade serous, endometrioid, and clear cell carcinoma organoids formed denser structures with multiple lumens. Moreover, the genomic features of each patient were well-preserved^28^, indicating that this biobank represented an excellent model for investigating ovarian cancer intra- and inter-patient heterogeneities.

### The glioblastoma organoid biobank

Glioblastoma is the most common brain tumor in adults and is characterized by elevated invasiveness^77^. Heterogeneity between and within glioblastomas is the main reason for therapeutic resistance in clinical trials^78–80^. A recent report by Jacob et al.^73^ established a biobank consisting of 70 glioblastoma organoids from 53 patients. Transcriptome sequencing, whole-exome sequencing, and single cell transcriptome sequencing showed that intra- and inter-tumor heterogeneities are well maintained in glioblastoma organoids, reflecting the original tumors. A co-culture system including glioblastoma organoids and chimeric antigen receptor T (CAR-T) cells was used to determine the CAR-T cell treatment response, suggesting a possible application in personalized therapy^73^.

### The liver cancer biobank

Broutier et al.^29^ first established primary liver cancer organoids in 2017. They included HCC, CC, and CHC subtypes from 8 surgically resected liver tumor tissues and were initially called “tumoroids”. Based on this culture method, Nuciforo et al.^65^ reported the generation of 13 patient-derived liver cancer organoids using 10 HCC needle biopsies from 8 patients and 3 intrahepatic cholangiocarcinoma needle biopsies from 3 patients. They demonstrated the replication of morphology and genetic heterogeneities of the original tumors^65^. Furthermore, in 2019, Li et al.^74^ generated 27 liver cancer organoid lines and tested them with 129 FDA-approved cancer drug libraries, illustrating the potential of cancer organoids in drug discovery. However, large-scale liver cancer organoid biobanks have not yet been reported.

## Tumor organoids can be generated from genetically modified normal organoids

Along with organoids directly derived from patients diagnosed with carcinomas, organoids derived from non-cancerous tissue can also be used to establish biobanks for cancer research *via* genetic modification. Indeed, this method has been reported to mimic several carcinomas. Huang et al.^23^ differentiated human pluripotent stem cells into exocrine progenitor organoids *via* 3D cultures. *KRAS* or *TP53* mutation-specific cancer organoids were successfully generated to model PDAC, and precision therapy strategies were identified. Furthermore, mutations in *APC*, *SMAD4*, and *PIK3CA* were edited by CRISPR/Cas9 technology in non-cancerous colon organoids to create colorectal carcinomas, illustrating the production of malignant phenotypes^68,81^.

CRISPR-based genetic modifications provide important clues for cancer initiation. Sun et al.^82^ used genetically reprogrammed human hepatocyte organoids to mimic the initial alterations in liver cancers. They found that c-Myc expression led to a malignant HCC morphology in these hepatocytes, whereas *RAS^G12V^*, *YAP^5SA^*, *IDH2^R172K^*, and *PTPN^3A90P^* organoids developed morphological alterations similar to intrahepatic cholangiocarcinomas, indicating a tractable system in cancer modeling^82^.

CRISPR-based genetic modification enables the investigation of differentiated cancer subtypes and provides vital tools for tracing cancer progression. Seino et al.^64^ performed *GATA6* short hairpin RNA-based knockdown and CRISPR-Cas9-based knockout experiments in Wnt-non-secreting PDAC organoids. They found that *GATA6* expression levels were functionally relevant in PDAC subtypes^64^. Moreover, CRISPR-Cas9-based genomic editing of PDAC driver genes in organoids, such as *KRAS*, *TP53*, *CDKN2A*, and *SMAD4*, demonstrated non-genetic acquisition of Wnt niche independence during pancreatic cancer initiation and progression^64^.

Together, genetic modifications can accelerate the establishment of large-scale tumor organoid biobanks while allowing for the characterization of tumor initiation and progression.

## Organoids preserve tumor heterogeneity of the original patient

Tumor heterogeneity is a common feature of multiple cancers. It can be divided into intra- and inter-tumor heterogeneities, and plays a vital role in tumor progression, tumor response to therapy, and the emergence of drug resistance^83,84^. Traditional cell culture and PDX models are not capable of replicating the heterogeneity of the original patient^85^. As a result, organoid models have been introduced to investigate cancer heterogeneity.

Organoids replicate the genomic profile of the original patient. For example, Wetering et al.^70^ performed WGS to investigate the heterogeneity of colorectal cancer in the biobank they established. They reported alterations in tumor suppressors, such as *APC*, *FBXW7*, *TP53*, and *SMAD4*, and activating mutations in *KRAS* and *PIK3CA*, which were consistent with previous reports on primary colorectal carcinomas^70^. Lee et al.^30^ demonstrated that bladder cancer organoids showed >80% concordance with the mutational profiles of parental tumors, such as mutations in *TP53* and *RB1* or the *FGFR3-TACC3* fusion. Kopper et al.^28^ performed WGS in 40 ovarian cancer organoids from 22 patients and found that most copy number variants, somatic single nucleotide variants, and structural variants were preserved in cancer organoids, consistent with the original tumors, even after prolonged passaging. Broutier et al.^29^ demonstrated that ∼92% of the variants of each patient were retained in early organoid cultures (<2 months) and >80% of variants were maintained in late organoid cultures (>4 months), further suggesting that the genomic profiles of the original patients were preserved in cancer organoids.

Organoids replicate the transcriptome profiles of original patients. Yan et al.^72^ performed transcriptomic analyses of gastric cancer organoids derived from different histological subtypes and counterpart tumor tissues, and demonstrated a high concordance of gene expressions. In particular, several genes and pathways appeared upregulated in cancer organoids, such as the HOX family, the mitotic cell cycle, and Wnt pathway-associated genes^72^. Broutier et al.^29^ compared expression profiles using RNA sequencing and demonstrated that each organoid correlated with its corresponding tissue of origin. HCC markers, such as *GPC3* and *AFP*, and hepatocyte markers, such as *ALB*, *TTR, APOA1*, and *APOE*, were highly expressed in HCC organoids and matched tissues; whereas CC and ductal markers, such as *EPCAM*, *KRT19*, and *S100A11*^86^, were highly expressed in CC organoids, which was consistent with the characteristics of clinical primary liver cancers^29^.

Cancer heterogeneity is largely maintained within organoids at different time points. Kopper et al.^28^ used a novel single cell DNA sequencing method to investigate recurrent ovarian cancer tumor samples derived from a single patient at different time points and the corresponding organoids. Distinct clusters revealed by copy number variant profiles overlapped with each other and did not generate new separate clusters, suggesting both their heterogeneity and resemblance to the corresponding tumor samples^28^. Notably, in some lines, cluster-represented diploid cells became fewer after passaging, indicating the gradual overgrowth of tumor cells compared with noncancerous cells, while maintaining tumor heterogeneity^28^.

Organoids mimic the evolution of heterogeneity both *in vitro* and *in vivo.* Heterogeneity evolution is a universal phenomenon in cancer^87^, which was challenging to manipulate in patients or xenografts before the application of effective organoid tools. Lee et al.^30^ performed deep sequencing to compare the mutational profiles of organoids in culture, during grafting or when re-establishing organoid lines from grafted models. They found that mutational profiles were largely maintained within individual lines, while a small subset of mutations underwent evolution. By analyzing the clonal composition of each line, they found that truncation mutations were retained, whereas subclonal mutations could be gained or lost, such as a gain of *CTNNB1^S45F^* in late passage organoids and losses of *ERBB2^D227N^* and *JAK2^H538Y^* during organoid culture, confirming clonal evolution during serial passaging^30^. Therefore, organoids can be used to investigate heterogeneous evolution in cancer.

Based on heterogeneity, organoids can contribute to the identification of potential prognostic biomarkers in carcinomas. Broutier et al.^29^ compared the similarities between transcriptomes of all primary liver cancer organoids to healthy liver-derived organoids and identified previously unknown genes. Specifically, overexpression of *C19ORF48*, *DTYMK*, or *UBE2S* in HCC and overexpression of *C1QBP* in CCs conferred poor survival prognoses^29^. This example highlighted the important role of organoid heterogeneity in identifying new targets with prognostic value, which could potentially be used in clinical cancer therapy.

## Organoids in drug screening and personalized medicine

The lack of tumor models suitable for drug screening has shown the need for systems more representative to those in patients, which are more amenable to disease modeling and individual regimen design^88^. In recent years, organoid biobanks of multiple cancers have been used to mimic tumor heterogeneity of the original patients.

Based on tumor heterogeneity, organoids can be used to test drug efficiency in both clinical therapy and clinical trials. Wetering et al.^70^ found that organoids derived from the same patient, even those carrying the BRAF V600E mutation, differed in their drug response profiles, emphasizing the importance of drug screening based on tumor heterogeneity. Sachs et al.^27^ found that breast cancer organoids were sensitive to drugs that blocked the HER signaling pathway when *HER2* was overexpressed or when other drug resistant genes existed in the absence of *HER2*. In addition, breast cancer organoid lines characterized by high BRCA1/2 signatures were sensitive to poly (ADP-ribose) polymerase inhibitors; whereas those with low BRCA1/2 signatures were not. These discordant drug efficacies were all based on the presence of different subpopulations^27^. Broutier et al.^29^ used liver cancer-derived organoids to screen 29 anti-cancer compounds, including drugs in clinical use or development. Notably, correlations between drug sensitivities and molecular profiling were observed. For example, HCC organoids harboring a mutated *CTNNB1* were resistant to the porcupine inhibitor LGK974; whereas the Wnt-dependent organoid line of CC was sensitive. Wild-type *KRAS* organoid lines of HCC were sensitive to the EGF receptor inhibitor AZD8931, while organoids harboring mutated *KRAS* were resistant, suggesting guidance for personalized medicine based on tumor heterogeneity^29^.

Organoids can also be used to predict the drug response. Sachs et al.^27^ compared the response of breast cancer organoids to tamoxifen in patients administered standard clinical treatment and found that *in vitro* drug responses of breast cancer organoids matched those of the corresponding patients, indicating the potential use of organoids in predicting *in vivo* drug response^27^. Vlachogiannis et al.^33^ compared the response of organoids treated with anti-cancer agents with that of patients in clinical trials. Accordingly, organoids showed 100% sensitivity, 93% specificity, 88% positive predictive value, and 100% negative predictive value in forecasting the response to targeted agents or clinical chemotherapy, thus illustrating the ability of organoids to predict the drug response in personalized medicine.

Organoids can also be used for large-scale drug and toxicology screenings. Skardal et al.^88^ summarized the development of organoid-on-a-chip platforms. In this system, organoids generated from multiple cancer cells were encapsulated in Matrigel in separate chambers, while myeloblasts or fibroblasts were formed with alginate gels in additional chambers to mimic the tumor microenvironment^89^. These organoids-on-a-chip platforms supported organoid culture, fluid flow, and high-throughput testing, offering the potential for drug screening while taking into account a personalized tumor microenvironment^88^.

Importantly, organoids can be used for modeling personalized immunotherapy^50^. Immunotherapy in tumors has not been very effective and remains to be further developed^52^. With the improvement of tumor-immune co-cultured organoid systems, Fadi Jacob et al.^73^ co-cultured glioblastoma organoids with CAR-T cells designed to react specifically with cells expressing EGFRvIII. By testing the proliferation of T cells and death of tumor cells in the presence of EGFRvIII in this system, they demonstrated the utility of organoids for rapid testing of immunotherapy with the endogenous target in culture^73^. In addition, organoids can serve as a platform for investigating the functional response to anti-PD-1 or anti-PD-L1^52^. Neal et al.^52^ established 20 organoids representing the immunotherapy-responsive neoplasms and treated organoids with the therapeutic PD-1 blocking antibody, nivolumab. As a result, nivolumab elicited significant induction of IFNG, PRF1 and/or granzyme B within organoid CD3+ tumor-infiltrating lymphocytes, showing functional *in vitro* recapitulation of checkpoint inhibition, which was consistent with personalized anti-PD-1 responses in clinical trials.

In addition, patient-derived organoid models can capture patient specific tumor evolution and acquired resistance to treatment^33^. Vlachogiannis et al.^33^ generated organoids using liver metastasis tissues from a colorectal cancer patient both before and after regorafenib treatments, which were used to establish xenografts. Consistent with clinical findings, CD31 immunostaining revealed a significant reduction in microvasculature in response to regorafenib in xenografts established with nontreated organoids, while no significant change was observed in xenografts established using regorafenib-treated organoids. More importantly, regorafenib treatment provided a selective survival benefit in mice with nontreated organoids^33^, suggesting the predictive role of organoids in reflecting cancer evolution upon treatment.

Another application of organoids in personalized medicine is the ability to target specific tumor cells while leaving healthy cells unharmed^11^. Drost and Clevers suggested the application of hepatocyte organoids in testing for hepatotoxicity, one of the leading causes of drug failure in clinical cancer therapy. They hypothesized that a suitable drug killed only tumor organoids without inducing hepatotoxicity^11^. Drug nephrotoxicity is another crucial cause of therapy failure in hospitalized patients^90^. By using organoids to demonstrate the toxicity of cisplatin in the proximal and distal tubules of the kidney, Morizane et al.^91^ established an important breakthrough in studies on nephrotoxicity. Furthermore, cardiac organoids derived from induced pluripotent stem cells have also been used to investigate cardiotoxicity^92,93^. These organoids provide patient-specific models for studying the toxicity of anti-cancer drugs in personalized medicine.

## Challenge

Despite the advantages of organoids, there are still shortcomings compared with other models^50^. For example, organoids derived from tissue pieces indeed capture interpatient genetic heterogeneities and contain all cellular components of the tissue microenvironment; however, several organoid cell lines, especially tumor-immune co-cultured organoid systems, might show limited culture times^94,95^ when compared with cancer cell lines or PDX models. In addition, organoids generated from pluripotent stem cells showed the ability of serial passaging while not containing all cellular components of the tissue microenvironment, including fibroblasts, endothelial cells, and immune cells, when compared with the PDX models^95,96^. It is therefore important to determine whether organoid models are most appropriate in investigating tumor biology in specified conditions.

Although organoids derived from most carcinomas have been established, efforts still need to be made to improve the organoid model system. First, it is indispensable to reduce the cost of cultivating organoids to ensure their wider applicability. Gjorevski et al.^97^ replaced mouse-derived extracellular matrices with engineered synthetic matrices in mouse and human intestinal organoid culture systems. The new matrices still supported organoid growth and expansion, but at substantially reduced culture costs^97^, thus stimulating the search for alternatives to other ingredients employed in organoid cultures^63^. Organoids derived from several advanced cancers grow more slowly than those derived from non-cancerous epithelium, which may result in the outgrowth of healthy tissue and hinder cancer research. Hence, separating cancer organoids from noncancerous organoids remains a challenge^58^. In addition, vascular and neural systems supporting cancer growth and metastasis are not included in current organoid models^58^. However, a functional enteric nervous system was developed in intestinal organoids using induced pluripotent stem cell-derived tissue engineering, indicating the potential for developing more complex structures within organoids^98^. The introduction of vascular and neural systems and advanced structures in organoids will help replicate the tumor microenvironment to assess its role in cancer.

## Outlook

In recent years, the rapid development of organoid platforms has enabled the successful establishment of cancer biobanks. Furthermore, CRISPR-based genetic modifications have allowed the exploration of cancer initiation and progression using organoid models. Based on genomic and transcriptomic characterizations, original intra- and inter-patient heterogeneities associated with a wide range of carcinomas have been successfully preserved in cancer organoids. Organoids are therefore predicted to play a decisive role in drug screening and personalized medicine, which will provide vital guidance for clinical cancer therapy.
